# Safety and Feasibility of Lung Cancer Surgery under the COVID-19 Circumstance

**DOI:** 10.3390/cancers14051334

**Published:** 2022-03-04

**Authors:** Lawek Berzenji, Leonie Vercauteren, Suresh K. Yogeswaran, Patrick Lauwers, Jeroen M. H. Hendriks, Paul E. Van Schil

**Affiliations:** Department of Thoracic and Vascular Surgery, Antwerp University Hospital, Drie Eikenstraat 655, 2650 Edegen, Belgium; lawek.berzenji@uza.be (L.B.); leonie.vercauteren@uza.be (L.V.); sureshkrishan.yogeswaran@uza.be (S.K.Y.); patrick.lauwers@uza.be (P.L.); jeroen.hendriks@uza.be (J.M.H.H.)

**Keywords:** lung cancer, COVID-19, SARS-CoV-2, surgery, safety, feasibility, guidelines

## Abstract

**Simple Summary:**

The global coronavirus disease 2019 (COVID-19) pandemic has drastically changed the current practice of medicine worldwide. As more clinical data is collected and processed, we are beginning to have an understanding of which patients are more at risk for severe complications of severe acute respiratory syndrome coronavirus 2 (SARS-CoV-2) infections. Preliminary data has shown that patients with lung cancer are disproportionally affected by the current COVID-19 pandemic. Furthermore, studies have shown that lung cancer patients are also significantly more likely to be admitted to the ICU and need mechanical ventilation. A specific subset of patients that are even more at risk for severe COVID-19 are those that require lung cancer surgery. To minimize the risk of SARS-CoV-2 infections in patients undergoing surgery, new treatment guidelines and preventive measures are necessary. In this review, we summarize the latest evidence regarding recommendations for patients undergoing lung cancer surgery in the COVID-19 era.

**Abstract:**

The current coronavirus disease 2019 (COVID-19) pandemic has forced healthcare providers worldwide to adapt their practices. Our understanding of the effects of COVID-19 has increased exponentially since the beginning of the pandemic. Data from large-scale, international registries has provided more insight regarding risk factors for severe acute respiratory syndrome coronavirus 2 (SARS-CoV-2) infections and has allowed us to delineate specific subgroups of patients that have higher risks for severe complications. One particular subset of patients that have significantly higher risks of SARS-CoV-2 infection with higher morbidity and mortality rates are those that require surgical treatment for lung cancer. Earlier studies have shown that COVID-19 infections in patients that underwent lung cancer surgery is associated with higher rates of respiratory failure and mortality. However, deferral of cancer treatments is associated with increased mortality as well. This creates difficult situations in which healthcare providers are forced to weigh the benefits of surgical treatment against the possibility of SARS-CoV-2 infections. A number of oncological and surgical organizations have proposed treatment guidelines and recommendations for patients planned for lung cancer surgery. In this review, we summarize the latest data and recommendations for patients undergoing lung cancer surgery in the COVID-19 circumstance.

## 1. Introduction

The global coronavirus disease 2019 (COVID-19) pandemic and its resultant strain on healthcare systems have drastically changed the current practice of medicine worldwide [1,2,3]. As more and more data from large-scale observational studies emerge, our understanding of the impact of severe acute respiratory syndrome coronavirus 2 (SARS-CoV-2) is growing exponentially [4]. We have seen that the disease course of patients that have contracted SARS-CoV-2 infection is phenotypically diverse. A large number of patients present with only mild respiratory symptoms or none at all. However, some patients can develop very severe symptoms, including acute respiratory distress syndrome (ARDS), cytokine storms, and multi-organ dysfunction [5,6,7]. Since the beginning of the COVID-19 pandemic, researchers have attempted to delineate subgroups of patients that are more at risk of severe morbidity and mortality. Several risk factors have already been identified, including advanced age, male sex, diabetes, obesity, cardiovascular disease, underlying pulmonary disease, and numerous other medical conditions [8,9,10,11]. Data has also shown that patients with underlying malignancies have higher mortality rates, which estimates ranging between 3.7 and 61.5% [12,13]. For lung cancer, previous studies have demonstrated that lung cancer patients have higher risks of contracting COVID-19 and are significantly more likely to develop severe (respiratory) symptoms, with an estimated mortality rate of 30–50% [14,15,16,17]. However, despite these increased risks, the provision of quality care for cancer patients remains vital [18]. In the majority of countries all over the world, this has not been an easy task. Many major cities with large hospital networks became epicenters for the COVID-19 pandemic, resulting in large-scale shortages of medical equipment, drugs, intensive care unit (ICU) beds, and trained medical staff. In addition, hospitals have been forced to change their procedures, set up new protocols, and reorganize their wards, all in a very short time and with minimal interference to their non-COVID healthcare [19,20].

One particular subset of cancer patients that may be at higher risk for severe COVID-19 are those that require lung cancer surgery for non-small cell lung cancer (NSCLC). Earlier data has shown that COVID-19 in patients with NSCLC that underwent lung resection is associated with higher likelihood of respiratory failure and mortality [21,22]. Currently, there is no conclusive, long-term data on which lung cancer surgery procedures should be prioritized and which can be deferred. In addition, there is no consensus on the possible impact of delaying surgical procedures for patients with NSCLC [23]. However, data analysis of the US National Cancer Database has shown that intervals longer than 6–12 weeks between initial diagnosis of early-stage lung cancer and surgical treatment result in lower survival rates compared to shorter intervals [24]. To aid clinicians in prioritizing surgical treatments, several institutes such as the American College of Surgeons and the European Society for Medical Oncology (ESMO) have developed guidelines for patient selection regarding surgical procedures [25,26]. In addition to the problem of patient prioritization, it is also still unclear whether risk mitigation measures are necessary for patients undergoing lung cancer surgery and, if so, what these measures should constitute. Numerous protocols have been proposed, often varying per country or even per hospital, thus creating heterogeneity in the care for patients undergoing lung cancer surgery [27]. In this review, we aim to summarize the latest data on COVID-19 and its associated risks for lung cancer patients undergoing surgery. Furthermore, we will discuss the currently available data on the safety and feasibility of lung cancer surgery in the current pandemic.

## 2. Lung Cancer and COVID-19

### 2.1. Epidemiology of COVID-19 in Lung Cancer Patients

An increasing number of studies have shown that cancer patients are disproportionally affected by the current COVID-19 pandemic [5,15,28]. It is estimated that between 1 and 8% of all patients hospitalized with COVID-19 have a history of solid tumors or hematological malignancies [29]. Furthermore, the available data also suggests that cancer patients are also significantly more likely to develop severe complications, generally represented by admission to the ICU and a need for mechanical ventilation [12,22]. This higher likelihood of developing a severe course of COVID-19 is also observable in the mortality rates: estimated mortality for patients with cancer range between 8 and 30%, depending on the type and stage of the malignancy [30]. Although it is not clear exactly why patients with cancer are more vulnerable to SARS-CoV-2 infections and which mechanisms are involved, several hypotheses have been proposed. It is likely that cancer patients are more prone to SARS-CoV-2 infections due to a combination of factors, such as frequent visits to hospitals and contacts with healthcare providers, advanced age or comorbidities, an immunocompromised state as a result of the cancer itself or cancer treatments, or due to additional medical therapies such as corticosteroids [30,31].

### 2.2. Morbidity and Mortality of COVID-19 in Lung Cancer

For lung cancer patients and patients with hematological malignancies, the risk of mortality due to SARS-CoV-2 infections is the highest among all cancer patients [30,32]. In the majority of patients with hematological cancers, this is due to prolonged periods of immunosuppression, something that is not typically seen in patients with lung cancer. Instead, the high morbidity and mortality rates of COVID-19 in lung cancer patients is mainly due to several pathophysiological, clinical, and treatment-associated factors [33]. In fact, it has been reported that patients with lung cancer in each group, subtype, and pathological tumor stage are more susceptible to infections with SARS-CoV-2 [34]. One of the major factors that has been correlated with higher incidence rates and severity of SARS-CoV-2 infections is the smoking history. This phenomenon has also been proven in the setting of influenza, in which smokers are five times more likely to contract influenza compared to nonsmokers [35]. In a systematic review by Vardavas et al., the authors evaluated the associated between smoking and outcomes of COVID-19. Their data showed that smokers have a 1.4x higher risk of developing severe symptoms compared to nonsmokers (RR = 1.4; 95% CI: 0.98–2.00). In addition, the risk of ICU admission, mechanical ventilation, and mortality was 2.4× higher in smokers (RR = 2.4; 95% CI: 1.43–4.04) [36]. This increased susceptibility to infections in smokers is mainly due to structural and immunologic-induced changes in the lungs. Smoking induces peribronchiolar inflammation and fibrosis, which facilitates the adherence of pathogens that can lead to increased rates of lung inflammation [37]. Furthermore, cigarette smoke also has a significant effect on the immune system—both proinflammatory and immunosuppressive. The proinflammatory effects of smoking have been documented extensively in earlier studies. Cigarette smoke induces the production of a number of proinflammatory cytokines, such as tumor necrosis factor alpha (TNF-α), interleukin (IL-)1, IL-6, IL-8, and granulocyte-macrophage colony-stimulating factor (GM-CSF). Furthermore, it amplifies the accumulation of immune cells in the airways [38,39]. However, cigarette smoke seems to have immunosuppressive properties as well, which seems to be predominantly caused by nicotine. Data from in vitro studies has shown that nicotine decreases the production of IL-6, IL-8, and IL-10 [40]. Furthermore, one of the major pathways resulting in immunosuppression is associated with the activation of α7 nicotinic acetylcholine receptor on macrophages, T cells, and B cells. This combination of inflammation-induced lung tissue damage, and an inadequate immune response is likely one of the major predisposing factors for severe complications of COVID-19 in smokers [41]. In addition, patients with prior tobacco-related lung damage, including chronic obstructive pulmonary disease (COPD), also have a higher cumulative risk for severe COVID-19 injury [42].

Aside from tobacco-related damage, lung cancer has been shown to be an independent risk factors for COVID-19 morbidity and mortality as well [43]. Several important genes commonly involved in cancer have been associated with severe SARS-CoV-2 infections [44]. ACE2 is a cellular receptor for the viral entry of SARS-CoV-2 particles and is widely expressed in the lungs. These viral particles use ACE2 receptors to fuse with the host cell membrane or to enter via endocytosis, a process regulated by type II transmembrane serine proteases such as TMPRSS2. In patients with lung cancer, both the TMPRSS2 and ACE2 levels are significantly elevated, suggesting an increased risk for SARS-CoV-2 infections [45]. Another gene that is both common to lung cancer and COVID-19 is the plasminogen activator inhibitor 1 (PAI-1) gene, which encodes PAI-1, a member of the serine protease inhibitor family. PAI-1 functions as the principal inhibitor of tissue plasminogen activator (tPA) and urokinase (uPA). Both SARS-CoV-2 and lung cancer induce elevated levels of PAI-1, which can result in the formation of thrombosis. Other SARS-CoV-2 related genes, such as basigin and furin, have been identified as well, and their roles in the pathophysiology of lung cancer and COVID-19 are being investigated [46].

### 2.3. Tumour Microenvironment

One important hypothesis as to how lung cancer increases the risk for severe COVID-19 disease is that the SARS-CoV-2 is able to modulate the lung tumor microenvironment (TME). The TME is the immediate surrounding environment of a primary tumor or metastasis and is mainly composed of stromal cells such as T-cells, B-cells, natural killer (NK) cells, fibroblasts, adipocytes, vascular endothelial cells, and pericytes. In general, these cells secrete signals that allow tumors to survive, grow, invade, and migrate, a process often referred to as oncomodulation [47]. Earlier data has shown that a number of factors, such as smoking, inflammation mediators, and infections, can modulate the TME, making it more tumor-friendly. In lung cancer patients, the TME provides a lung environment that is suitable for virus entry, duplication, and infection. Indeed, several studies have already shown that SARS-CoV-2 proteins can modulate the lung TME through disruption of the already fragile immune mechanisms in the lung. These processes can lead to a severe cytokine response, also referred to as a cytokine storm, and the formation of thrombi due to alveolar damage and fibrin deposits. This massive cytokine release and the formation of thrombi are two of the most important pathogenic pillars associated with the development of ARDS in patients with SARS-CoV-2 infections [48].

### 2.4. Other Risk Factors

There are several other factors that could contribute to higher risks for SARS-CoV-2 infections in lung cancer patients. Advanced age is a well-known factor that is associated with an increased risk of mortality in COVID-19 patients, with mortality rates steadily rising from 65 years onwards. This age group also encompasses the majority of patients with lung cancer [5]. In addition to advanced age, concomitant medications such as corticosteroids and antineoplastic therapies can likely increase the susceptibility for SARS-CoV-2 infections in lung cancer patients [30,49]. A large proportion of lung cancer patients receive corticosteroids as prophylactic or therapeutic treatment for symptoms of cancer or COPD. However, treatment with steroids can mask early signs of infections and can lead to a delayed diagnosis of SARS-CoV-2 infection in patients with lung cancer [50]. Another hotly debated topic is the use of immunosuppressive therapies such as chemotherapy, immunotherapy, and targeted therapies during the COVID-19 pandemic. Recent evidence has shown that chemotherapy within the month preceding a diagnosis of SARS-CoV-2 was associated with significantly higher complication rates [33]. For immunotherapy and targeted therapies, the case is less clear, and data is lacking. In a multicenter, retrospective study by Aschele et al., 59,989 patients receiving antitumor treatment for lung cancer were analyzed for SARS-CoV-2 infections. Their data showed that a total of 406 patients (0.68%) developed COVID-19. The authors concluded that antitumor treatments are associated with low probabilities of SARS-CoV-2 infections [51].

## 3. Lung Cancer Surgery in the COVID-19 Era

### 3.1. Early Phase of COVID-19 Pandemic

The main goal of the treatment of patients with lung cancer during the COVID-19 pandemic is to provide high-quality oncological care while simultaneously minimizing the risks of contracting SARS-CoV-2 or the risk of severe complications should they develop COVID-19 [52]. In the early phase of the pandemic, many hospitals were forced to delay surgical procedures due to shortages of personal protective equipment (PPE) and ICU beds and overwhelming numbers of COVID-19 patients [53]. At that time, there was limited data available regarding the management of non-COVID-19 pathologies, and no clear guidelines had been proposed. For the surgical management of NSCLC, early guidelines often recommended multidisciplinary discussions on a case-by-case basis for the decision-making process [54]. Furthermore, some centers suggested postponing nonurgent surgical treatments for NSCLC or to consider nonsurgical treatment approaches using stereotactic body radiation therapy (SBRT), for example. This cautiousness mainly stemmed from the fact that mortality rates as high as 10–24% were reported for perioperative COVID-19 in the general surgical population [55]. Additionally, the UK Lung Cancer Coalition’s Clinical Advisory Group stated that patients receiving surgical treatment for NSCLC had increased mortality rates of 40–50% if they contracted SARS-CoV-2 infections following their surgical treatment for NSCLC [30,56]. However, as time passed, data from different single- and multicenter large-scale registries such as the Thoracic Cancers International COVID-19 Collaboration (TERAVOLT) and the COVID-19 and Cancer Consortium (CCC-19) provided more insight into which patients should be prioritized [30,57,58]. Table 1 shows an overview of international registries that have collected data on COVID-19 cases in patients with (lung) cancer. In addition, major oncological institutes such as the American Society of Clinical Oncology (ASCO), American College of Surgeons (ACS), and ESMO have published their evidence-based recommendations as well.

### 3.2. American College of Surgeons (ACS) Guidelines

The ACS COVID-19 guidelines are divided into three triage levels: a semi-urgent setting (phase I) in which there are few COVID-19 patients and sufficient hospital resources, an urgent setting (phase II) in which there are many COVID-19 patients with limited ICU capacity or hospital resources, and a highly urgent setting (phase III) in which (nearly) all hospital resources are routed to COVID-19 patients. In phase I, ACS recommends deferring NSCLC cases with predominantly ground glass nodules (<50% solid), solid nodules <2 cm, or cases with an indolent histology. Patients with (predominantly) solid lung cancers >2 cm and negative clinical nodes, patients with node positive NSCLC, and patients postinduction treatment need to be treated as soon as feasible. In phase II, the guidelines recommend deferring all elective thoracic procedures. Tumor-associated infections and surgical complications in hemodynamically stable patients should be treated at this time. Alternatively, healthcare providers can also choose to transfer patients to hospitals in phase I or to offer alternative treatments if possible, e.g., neoadjuvant therapy, SBRT, or definitive chemoradiation therapy (CRT). Finally, the ACS recommends for phase III that healthcare providers defer all cases except for patients with threatened airways, tumor-associated sepsis, or hemodynamically unstable patients with surgical complications [25]. The ASCO guidelines refer to the ACS recommendations for surgical treatment of lung cancer [19].

### 3.3. European Society for Medical Oncology (ESMO) Guidelines

**Figure 1:**
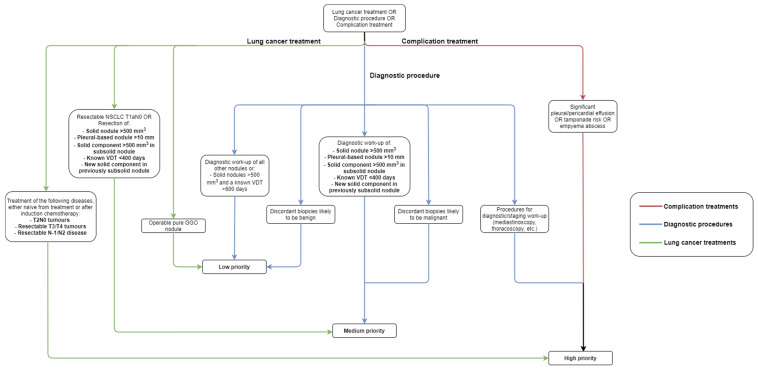
Flowchart showing different priorities for surgical management of patients with lung cancer (based on ESMO guidelines). GGO, ground glass opacity; NSCLC, non-small cell lung cancer; VDT, volume doubling time.

The ESMO guidelines for lung cancer surgery divide cases into three priority levels: high, medium, and low. High-priority procedures include drainage with or without pleurodesis of pleural effusion, treatment of pericardial effusions, and evacuation of pleural empyema. In addition, T2N0 disease, resectable T3/T4 tumors, and resectable N1/N2 disease naïve from treatment after neoadjuvant chemotherapy are all considered high priority as well. Finally, procedures including mediastinoscopy, thoracoscopy, biopsy, and endoscopy for diagnostic or staging purposes are high priority as well. Medium priority includes discordant biopsies that have high chances of being malignant and resectable T1aN0 disease. Furthermore, the diagnostic workup or surgical treatment of nodules with the following characteristics are considered medium priority as well: solid nodules or solid components in partially solid nodules >500 mm^3^, pleural solid nodules > 1 cm, a volume doubling time <400 days, or a new solid component in a previously nonsolid nodule. Low priorities include discordant biopsies that are likely to be benign, operable pure ground glass opacifications (T1A), and the diagnostic workup or resection of nodules >500 mm^3^ with a known volume doubling time >600 days. For medium and low priorities, the guidelines state that SBRT is an acceptable alternative treatment if surgical treatment is indicated when no surgical capacity is available [59]. However, it is important to note that recent data has suggested that delayed surgery for early-stage NSCLC is associated with improved survival outcomes compared to early treatment SBRT [60]. Figure 1 shows a flowchart based on the ESMO treatment guidelines. Table 2 shows a comparison between the ASC and ESMO guidelines.

### 3.4. Other Guidelines and Additional Measures

A few other noteworthy organizations that have published guidelines for lung cancer surgery are the British Thoracic Society (BTS), the International Association for the Study of Lung Cancer (IASLC), and the Thoracic Surgery Outcomes Research Network. The BTS and IASLC guidelines are less specific and offer more general recommendations for patients undergoing lung cancer surgery. In the guidelines of the BTS, the authors have given a set of general recommendations for the diagnosis and treatment of lung cancers. Regarding surgery, the guidelines recommend minimizing the hospital length of stay for patients planned for surgery by using minimally invasive approaches or day of surgery admission. Furthermore, patients with higher risks—more specifically, those not fit for a lobectomy—should be considered for referral for radiation therapy or radiofrequency ablation. In addition, surgeons may opt for surgical treatment without preoperative biopsy, using Herder score and frozen sections intraoperatively [61]. The IASLC guidelines summarizes the ACS and ESMO recommendations [52]. In the Thoracic Surgery Outcomes Research Network consensus statement, the authors also provide a three-phased guide similar to the ASCO recommendations for triaging patients [62].

In addition to these specific recommendations for patients undergoing surgery for NSCLC, the majority of guidelines include general guidance protocols as well. Patients planned for surgery should undergo reverse transcription polymerase chain reaction (RT-PCR) testing prior to treatment. If the COVID-19 test is positive, surgical treatment should be delayed for 2 to 3 weeks or until the patient is asymptomatic. All patients should be tested after their isolation period. Physicians and all operating room personnel should be screened daily for fever or COVID-19 symptoms. Healthcare providers with symptoms should be tested for COVID-19 and be quarantined for 2 weeks if the results are positive. In urgent surgical cases, procedures can be performed in specialized negative-pressure operating rooms. Surgeons and operating personnel should use full PPE. Postoperatively, patients should be treated in negative-pressure isolation rooms. Furthermore, the number of visitors should be drastically reduced or visitors should be prohibited altogether for patients after surgical treatment during their hospital stay [14,25,30,59,61,62].

**Table 1:** Overview of registries of COVID-19 cases in patients with lung cancer.

Study	Location	Study Dates	Study Characteristics	Study Definition of COVID-19	No. of Lung Cancer Patients	Mortality Rate	Lung Cancer Outcomes
MSKCC [63]	New York, USA	12 March–6 May 2020	Single-center, retrospective study of patients with lung cancer and COVID-19	Positive SARS-CoV-2 RT-PCR test	102	25% (25/102)	COVID-19 was severe in patients with lung cancer (62% hospitalized, 25% died).
UKCCMP [53]	UK	18 March–8 May 2020	Single-center, retrospective study of patients with solid organ or haematological malignancies and COVID-19	Positive SARS-CoV-2 RT-PCR test	111	39% (43/111)	No increased case–fatality rate due to COVID-19 in lung cancer patients
OnCovid [64]	Multicenter	26 February–1 April 2020	Multicenter, retrospective study of patients with solid organ or haematological malignancies and COVID-19	Positive SARS-CoV-2 RT-PCR test	119	No specific data for lung cancer. Total mortality: 33.6%	Mean OS is 34.42 ±2.51 months (range 29.51–39.33)
GCO-002 [65]	France	1 March–11 June 2020	Single-center, combined retrospective and prospective study of solid organ tumours and COVID-19	Positive SARS-CoV-2 RT-PCR test; imaging features on chest CT; symptoms + serology	233	No specific data for lung cancer. Total thoracic cancer mortality: 30%	Thoracic malignancies associated with admission to ICU and/or mechanical ventilation and/or death (multivariate *p*-value = 0.04)
CCC-19 [17,57,66]	Multicenter	17 March–7 May 2020	Multicenter, retrospective study of patients with solid organ or hematological malignancies and COVID-19	Positive SARS-CoV-2 RT-PCR test	237	26% (61/237)	Lung cancer mortality is significantly higher than overall study mortality (26% vs. 16%)
TERAVOLT [23,58,67]	Multicenter	26 March–15 July 2020	Multicenter, cross-sectional retrospective study of thoracic malignancies	Positive SARS-CoV-2 RT-PCR; clinical diagnosis (symptoms and contacts); imaging features on chest CT	1012	32% (326/1012)	Hospitalization rate: 72% (733/1012); ICU admission: 12% (118/1012); Mechanical ventilation: 25% (248/1012)

COVID-19, coronavirus disease 2019; CT, computed tomography; ICU, intensive care unit; OS, overall survival; RT-PCR, real-time polymerase chain reaction; SARS-CoV-2, severe acute respiratory syndrome coronavirus.

**Table 2:** Comparison between ACS and ESMO guidelines for lung cancer surgery during the COVID-19 pandemic.

**Guideline Topic**	**ACS**	**ESMO**
Triaging system	Based on number of COVID-19 patients, hospital resources and ICU/ventilator capacity	Based on disease factors (e.g., TNM stage, symptoms) and type of procedure
High urgency	- Threatened airway - Tumor-associated sepsis - Surgical complications in hemodynamically unstable patients (active bleeding requiring surgical management, airway dehiscence, anastomotic leak with sepsis)	- Drainage with(out) pleurodesis of pleural effusion, pericardial effusion, tamponade risk - Empyema-abscess - Untreated T2N0 tumors or after induction chemotherapy - Resectable, untreated T3/T4 tumors or after induction chemotherapy - Resectable, untreated N-1/N2 disease or after induction chemotherapy - Diagnostic procedure such as mediastinoscopy/thoracoscopy/pleural biopsy/endoscopy/transthoracic investigations for diagnostic/staging workup
Medium urgency	- Tumor associated infection: compromising, but not septic (e.g., debulking for post-obstructive pneumonia) - Management of surgical complications (hemothorax, empyema, infected mesh) in a hemodynamically stable patient	- Discordant biopsies likely to be malignant - Resectable NSCLC with T1AN0 (alternative if no surgical capacity available, is SBRT; surgery is preferred) - Diagnostic workup and/or resection of nodules of incidental finding with either: Solid nodule > 500 mm^3^ Pleural-based solid nodule > 10 mm Solid component > 500 mm^3^ in partially solid nodule Known VDT < 400 days New solid component in pre-existing non-solid nodule
Low urgency	- (Predominantly) solid (>50 percent) lung cancer or presumed lung cancer > 2 cm, clinically node-negative - Node+ lung cancer - Post-induction therapy - Staging to start treatment (mediastinoscopy, diagnostic VATS for pleural dissemination) - Patients enrolled in therapeutic clinical trials	- Discordant biopsies likely to be benign - Operable pure GGO nodule (T1a) - Diagnostic workup and/or resection of all other nodules of incidental finding including also: Solid nodule > 500 mm^3^ and known VDT > 600 days
Alternative treatment approaches recommendations (medium/high-urgency cases)	- Transfer patient to hospitals with more capacity - Administering neoadjuvant therapy for patients eligible for adjuvant therapy - SBRT - Ablation (such as cryotherapy, RFA) - Reconsider neoadjuvant as definitive chemoradiation, and follow patients for “local only failure” (i.e., salvage surgery)	- SBRT
Alternative treatment approaches recommendations (low-urgency cases)	- If eligible for adjuvant therapy, then give neoadjuvant therapy (for example chemotherapy for 5 cm lung cancer) - SBRT - Ablation (e.g., cryotherapy and RFA) - Debulking (endobronchial tumor) only in circumstance where alternative therapy is not an option due to increased risk of aerosolization (e.g., stridor postobstructive pneumonia not responsive to antibiotics) - Nonsurgical staging (endobronchial ultrasound, imaging, and interventional radiology biopsy) - Follow patients after their neoadjuvant for “local only failure” (i.e., salvage surgery) - Extending chemotherapy (additional cycles) for patients completing a planned neoadjuvant course	- SBRT

ACS, American College of Surgeons; COVID-19, coronavirus disease 2019; ESMO, European Society for Medical Oncology; GGO, ground glass opacities; ICU, intensive care unit; NSCLC, non-small cell lung cancer; RFA, radiofrequency ablation; SBRT, stereotactic body radiotherapy; TNM, tumor, node, metastasis; VATS, video-assisted thoracoscopic surgery; VDT.

## 4. Discussion

Since the beginning of the COVID-19 pandemic, healthcare providers worldwide have strived to offer non-COVID patients optimal treatments, despite the many strains the current pandemic has brought on our healthcare systems. In the case lung cancer patients, many endeavors have been made to ensure that patients receive treatments according to the latest therapeutic guidelines. For patients with early-stage or locally advanced NSCLC, surgical treatment is often recommended as primary modality or as treatment after induction chemotherapy. However, these patients have significantly higher risks for severe respiratory complications and mortality if they contract SARS-CoV-2, especially in the postoperative period [30,34,50,51]. A number of oncological and surgical organizations have proposed sets of recommendations and guidelines for triaging lung cancer patients planned for surgical treatment. These guidelines often divide patients into different groups in order to ensure that necessary and urgent treatments can be prioritized, even in regions where COVID-19 is rampant [25,52,59,61,62]. This allows healthcare providers to reduce the strain of the pandemic on their healthcare systems and to spread their elective procedures over extended periods, usually not longer than 6–8 weeks. In addition to these triaging protocols, a number of risk mitigation measures have been proposed as well. These measures often include recommendations regarding RT-PCR screening for patients and healthcare workers, quarantine periods, the use of PPE, and guidelines for surgeons during surgical treatment for patients without preoperative screening [14,25,52,54,59,61,62].

Several studies have investigated the safety and outcomes patients that underwent surgical resections for lung cancer. In a small retrospective study by Chang et al., 21 patients underwent pulmonary resection for lung cancer at NYU Langone Health between 13 March and 4 May 2020. Surgical treatments included 13 lobectomies, 6 segmentectomies, and 2 pneumonectomies. In this period, a total of 2087 COVID-19 patients were admitted to the same hospital as well. The authors investigated the safety of the patients that received surgical treatment, as well as healthcare provider safety, by using COVID-19 screening and by evaluating symptoms and early treatment outcomes. No major and five minor complications were observed in this patient cohort. The median hospital length of stay was 2 days. Furthermore, no previously COVID-19 negative patients or healthcare providers developed any symptoms or tested positive for SARS-CoV-2 infections in this period. The authors concluded that lung cancer surgery is safe, even if the surgical procedures are performed by healthcare providers who care for COVID-19 patients in hospitals with high rates of hospitalized COVID-19 patients [68]. In a similar study by Mejía et al., the incidence rates of SARS-CoV-2 infections and postoperative treatment outcomes were evaluated in patients that underwent thoracic surgery in a single center in Madrid (Spain) between 12 February and 30 April 2020. A total of 101 thoracic surgical procedures were evaluated, including 57 cases of pulmonary resection for lung cancer. Out of 57 oncological resections, five patients tested positive for COVID-19. However, all patients had a mild course of the disease without severe complications or ICU admission. The authors concluded that thoracic surgical procedures can be performed safely in selected patients and with strict COVID-19 protocols [69]. Another study worth mentioning was the study published by Leclère et al. Here, the investigators performed a retrospective analysis of a prospectively maintained multicenter database, including all patients that underwent surgical resection for NSCLC in Paris (France). The incidence rates and outcomes of COVID-19 after surgical resection of NSCLC were evaluated. A total of 115 patients with a mean age of 64.6 ± 10.7 years were included and analyzed. Their data showed that six patients (5%) tested positive for COVID-19 in the first month after surgery. Interestingly, all COVID-19-positive patients were operated during the first month of the pandemic. Furthermore, COVID-19 positivity was significantly associated with preoperative treatment with corticosteroids (*p* = 0.03) and with increased rates of readmission (*p* = 0.004). There were no significant differences in 30-day morbidity or mortality rates. In addition, no differences were observed regarding oncological outcomes such as R0 resections, rates of nodal upstaging, or need for adjuvant chemotherapy [54]. Other case series have shown similar acceptable outcomes of lung cancer surgery amidst the COVID-19 pandemic [68].

## 5. Conclusions

Despite our growing understanding of COVID-19 on the increasing numbers of studies regarding the effects of COVID-19 on our healthcare systems, many questions are still unanswered. For lung cancer surgery, the long-term epidemiological effects of treatment delays are yet to be seen. Furthermore, many patients have not visited hospitals despite symptoms due to concerns for contracting COVID-19 or due to healthcare providers discouraging them due to limited resources, resulting in an increasing number of preventable deaths. The aim of the numerous international guidelines and recommendations is to obviate this problem and to allow patients to receive the appropriate treatments that they need without excessive delays and in a safe manner. Furthermore, the collection of data by international registries such as TERAVOLT and CCC-19 will provide us with more insight into how (lung) cancer treatments should be tailored during this pandemic. The main hope of healthcare providers worldwide is that the vaccines will help slow the spread of COVID-19, allowing healthcare systems and hospitals to resume all treatments for non-COVID pathologies without further delays. Further research is still necessary to understand the pathophysiological mechanisms that lead to increased susceptibility and mortality rates of lung cancer patients that contract COVID-19. In addition, the safety of our surgical and nonsurgical treatments during COVID-19 surges need to be evaluated in larger, multicenter studies as well.

## Abbreviations

ACS	American College of Surgeons
ARDS	acute respiratory distress syndrome
ASCO	American Society of Clinical Oncology
BTS	British Thoracic Society
CCC-19	COVID-19 and Cancer Consortium
CI	confidence interval
CT	computed tomography
COPD	chronic obstructive pulmonary disease
COVID-19	coronavirus disease 2019
CRT	chemoradiation therapy
ESMO	European Society for Medical Oncology
GCO	Groupes Coopérateurs en Oncologie
GM-CSF	granulocyte macrophage colony-stimulating factor
IASCL	International Association for the Study of Lung Cancer
ICU	intensive care unit
IL	interleukin
MSKCC	Memorial Sloan Kettering Cancer Center
NK	natural killer
NSCLC	non-small cell lung cancer
OS	overall survival
PAI	plasminogen activator inhibitor
PPE	personal protective equipment
RR	relative risk
SARS-CoV-2	severe acute respiratory syndrome coronavirus 2
SBRT	stereotactic body radiation therapy
TERAVOLT	Thoracic Cancers International COVID-19 Collaboration
TME	tumor microenvironment
TNF	tumor necrosis factor
UKCCMP	UK Coronavirus Cancer Monitoring Project
